# Cytoprotective Effect of Hydroalcoholic Extract of *Pinus eldarica *Bark against H_2_O_2_-Induced Oxidative Stress in Human Endothelial Cells

**DOI:** 10.7508/ibj.2016.03.005

**Published:** 2016-07

**Authors:** Fatemeh Babaee, Leila Safaeian, Behzad Zolfaghari, Shaghayegh Haghjoo Javanmard

**Affiliations:** 1 Department of Pharmacology and Toxicology, Isfahan Pharmaceutical Sciences Research Center, School of Pharmacy and Pharmaceutical Sciences, Isfahan University of Medical Sciences, Isfahan, Iran;; 2 Department of Pharmacognosy, School of Pharmacy and Pharmaceutical Sciences, Isfahan University of Medical Sciences, Isfahan, Iran;; 3 Applied Physiology Research Center, School of Medicine, Isfahan University of Medical Sciences, Isfahan, Iran

**Keywords:** *Pinus eldarica*, Human umbilical vein Endothelial cells, Oxidative stress, Antioxidants

## Abstract

**Background:**

*Pinus eldarica* is a widely growing pine in Iran consisting of biologically active constituents with antioxidant properties. This study investigates the effect of hydroalcoholic extract of *P. eldarica *bark against oxidative damage induced by hydrogen peroxide (H_2_O_2_) in human umbilical vein endothelial cells (HUVECs).

**Methods:**

The total phenolic content of *P. eldarica *extract was determined using Folin-Ciocalteu method. The cytotoxicity of *P. eldarica *extract (25-1000 µg/ml) on HUVECs was assessed using 3-(4,5- Dimethylthiazol-2-yl)-2, 5-diphenyltetrazolium bromide (MTT) method. Cytoprotective effect of *P. eldarica *extract (25-500 µg/ml) on H_2_O_2_-induced oxidative stress was also evaluated by MTT assay. The intra- and extra-cellular hydroperoxides concentration and ferric reducing antioxidant power (FRAP) were measured in pretreated cells.

**Results:**

The total phenolic content of *P. eldarica *extract was estimated as 37.04±1.8% gallic acid equivalent. *P. eldarica* extract (25-1000 µg/ml) had no cytotoxic effect on HUVECs viability. The pretreatment of HUVECs with *P. eldarica *extract at the concentrations of 50-500 µg/ml significantly reduced the cytotoxicity of H_2_O_2_. *P. eldarica* extract decreased hydroperoxides concentration and increased FRAP value in intra-cellular fluid at the concentration range of 100-500 µg/ml and in extra-cellular fluid at the concentration range of 25-500 µg/ml.

**Conclusions:**

This study revealed the antioxidant and cytoprotective effects of *P. eldarica *extract against H_2_O_2_-induced oxidative stress in HUVECs. Concerning the high content of phenolic compounds in* P. eldarica*, more research is needed to evaluate its clinical value in endothelial dysfunction and in other oxidative conditions.

## INTRODUCTION

Oxidative stress, as a result of imbalance between the production of reactive oxygen species (ROS) and antioxidant defense, is implicated in the pathogenesis of various disorders such as cancer as well as neurodegenerative and cardiovascular diseases (CVDs)^[^^1^^,^^2^^]^. In vasculature, oxidative damage, which is induced by several stimuli, for instance, inflammation, ischemia and reperfusion, may cause endothelial dysfunction^[^^3^^]^. ROS can directly deactivate nitric oxide and reduce antioxidant capacity, thus contributing to vascular stress^[^^4^^]^. Oxidative stress has a causal role in developing CVDs (e.g. atherosclerosis, hypertension, and heart failure) via the oxidation of low-density lipoprotein, disruption of vascular function and induction of endothelial and myocardial cell apoptosis^[^^5^^,^^6^^]^.

Compounds or antioxidants with ability to inhibit the production of ROS or to scavenge ROS may offer therapeutic potential in the prevention or reduction of CVD. Recent studies have revealed some positive effects of antioxidant supplementation in improving endothelial function and modulating oxidative status^[^^7^^-^^9^^]^. Some investigations have also indicated that a high consumption of herbal antioxidants such as polyphenolics is associated with a reduced risk of CVD^[^^10^^,^^11^^]^. Besides, these antioxidants have shown to have anti-apoptotic effects on vascular endothelial cells following exposure to oxidizing agents like hydrogen peroxide (H_2_O_2_)^[^^12^^]^.

*Pinus eldarica *Medw. is a stout evergreen tree belonged to the family Pinaceae. This tree is widely growing in Iran with a common name of “Iranian pine” or “Tehran pine”^[^^13^^]^. In traditional medicine,* P. eldarica *is used to treat skin diseases, such as wounds, allergic rashes and dermatitis as well as bronchial asthma^[^^14^^,^^15^^]^. In a pharmacological study, *P. eldarica* bark extract has been revealed to have anti-hyperglycemic activity^[^^16^^]^. Phytochemical analysis of the fruits and bark oil of *P. eldarica *have demonstrated the presence of the biologically active constituents with antioxidant activities, including α-pinene, β-caryophyllene, β-pinene, longifolene, α-humulene, δ-3-carene, and junipene^[^^17^^,^^18^^]^. The high concentrations of polyphenolic compounds, e.g. catechin and taxifolin, have also been observed in P. eldarica bark extract^[^^19^^]^. 

The present study aimed to investigate the antioxidant and cytoprotective effects of hydroalcoholic extract of the *P. eldarica *bark against oxidative stress induced by H_2_O_2 _in human umbilical vein endothelial cells (HUVECs). We also evaluated the toxicity of *P. eldarica *extract in HUVECs to confirm the safety of this plant extract.

## MATERIALS AND METHODS

**Plant material and extract preparation**

The barks of *P. eldarica *were collected from Isfahan city, located in the Isfahan Province in the center of Iran during August 2014. After authentication of the plant by a botanist, a voucher specimen No. 3318 was deposited at the Herbarium of the School of Pharmacy and Pharmaceutical Sciences, (Isfahan, Iran) for future reference. To prepare hydroalcoholic extract, the powdered sample of air-dried barks of *P. eldarica *was extracted three times with ethanol (70%) using the maceration process at room temperature for 72 h. After the filtration of the extract, the solvent was removed using a rotary evaporator (Bibby RE200, UK) to produce a viscous brown residue, which was freeze-dried and stored at -20ºC. The yield of the plant extract was 21% (w/w). 

**Determination of total phenolic content**

The total phenolic content of *P. eldarica *extract was determined using Folin-Ciocalteu method. Briefly, sodium bicarbonate (20%) was added to the plant samples. The mixture was then treated with diluted Folin-Ciocalteu reagent. After 2 h, the absorbance was measured at 765 nm. The total phenol content was estimated using a standard curve obtained from various concentrations of gallic acid (50, 100, 150, 250, and 500 mg/l) and was expressed in the percentage of gallic acid equivalents^[^^20^^]^. 

**Cell culture**

HUVECs (National Cell Bank of Iran, Pasteur Institute of Iran, Tehran, Iran) were cultured in Dulbecco's modiﬁed Eagle's medium (GIBCO BRL, Life Technologies, Grand Island, USA) supplemented with 10% FBS (Bioidea Company, Tehran, Iran) and 1% penicillin-streptomycin (100 U/ml penicillin and 100 µg/ml streptomycin). HUVECs were incubated at a normal culture condition (95% humidified atmosphere of 5% CO_2_ at 37°C) in 25-cm^2^ and/or 75-cm^2^ flasks.

**Cell viability assay**

The probable cytotoxicity of *P. eldarica *extract on HUVECs was assessed by MTT method^[^^21^^]^ using a commercial kit (Bioidea Company, Tehran, Iran). In Brief, the cells were seeded at a concentration of 1.5×10^5^ cells/ml in 96-well plates. Twenty four hours after plating, the cells were treated with different concentrations of freshly prepared *P. eldarica *extract (25 to 1000 µg/ml) or vitamin C (100 µg/ml) and incubated at 37°C for additional 24 h. Then the medium of each well was removed. After washing the cells with PBS at pH 7.4, a fresh medium, and an MTT reagent were added to each well and incubated at 37°C for 3 h. MTT reaction with living cells produced insoluble foramazan crystals with purple color. After the addition of dimethyl sulfoxide for dissolution of formazan crystals, the absorbance was measured at 570 nm by a microplate reader (BioTek Instruments, PowerWave XS, Wincoski, USA).

The cytoprotective effect of *P. eldarica *extract on H_2_O_2_-treated HUVECs cells was evaluated by MTT assay. Following the exposure to the plant extract (25 to 500 µg/ml) or vitamin C (100 µg/ml), the cells were washed and treated with H_2_O_2_ (0.5 mM; Merck Co., Mumbai, India) for 2 h. 

The unexposed cells to the *P. eldarica *extract or H_2_O_2_ were considered as negative controls with the cell viability percentage of 100. Vitamin C-treated cells were used as the positive control. The viability of the treated samples was determined by a comparison between absorbance of various concentrations of the samples and negative control according to the following formula, and each experiment was performed in triplicate^[^^21^^]^. Cell viability (%)=(OD test-OD blank/OD negative control-OD blank)×100

**Measurement of intra- and extra-cellular hydro-peroxides concentration**

The effects of *P. eldarica *extract on intra- and extra-cellular hydroperoxides levels were determined based on the ferrous ion oxidation by xylenol orange reagent (FOX1)^[^^22^^]^. The FOX-1 reagent containing ammonium ferric sulfate in aqueous medium with sorbitol, was prepared according to the manufacturer’s protocol (Hakiman Shargh Research Co., Isfahan, Iran). After pretreatment with different concentrations of *P. eldarica *extract, HUVECs were exposed to H_2_O_2_. Then 10 μl supernatant of the cells or the cell lysates from each well was added to the 190-μl reagent and incubated at 40°C for 30 min. Absorbance was determined at 540 nm against a blank using a micro-plate reader/spectrophotometer (BioTek Instruments, PowerWave XS, Wincoski, USA). The hydroperoxides content of the samples were estimated using a standard curve of H_2_O_2 _concentrations (1-10 μM). 

**Measurement of cell-free and intra- and extra-cellular ferric reducing antioxidant power (FRAP)**

The total antioxidant capacity of different concentrations of *P. eldarica *extract was determined by the evaluation of FRAP^[^^23^^]^. FRAP value was measured based on the reduction of ferric-tripyridyltriazine complex to ferrous form by spectro-photometric assay. The FRAP reagent containing tripyridyltriazine/ferric chloride/acetate buffer was prepared according to the manufacturer’s protocol (Hakiman Shargh Research Co., Isfahan, Iran). For each well, 10 μl sample was added to the 200 μl FRAP reagent. The samples were supernatants of the cells or the cell lysates from each well^[^^24^^,^^25^^]^. FRAP assay was also carried out on samples without the cells. These samples were different concentrations of *P. eldarica *extract in water. The mixture of sample and reagent was incubated at 40ºC for 40 min. Then the absorbance was measured at 570 nm against the blank using a microplate reader (BioTek Instruments, PowerWave XS, Wincoski, USA). The FRAP values of the samples were calculated using the standard curve acquired from various concentrations of FeSO_4×_7H_2_O (0.1-10 mM) and were expressed as μM of FeII equivalents.

**Statistical analysis**

Data were presented as mean±standard error of mean (SEM). One-way analysis of variance (ANOVA) followed by Tukey's post-hoc test (SPSS software 

version 16.0) was used for statistical analysis. *P *value* <*0*.*05 was considered statistically significant.

## RESULTS

**Total phenolic content**

The total phenolic content was estimated as 37.04±1.8% gallic acid equivalents in dried barks of *P. eldarica* extract. 

**Effect of** ***P. eldarica*** **extract on human umbilical vein endothelial cells viability**

**Fig. 1:**
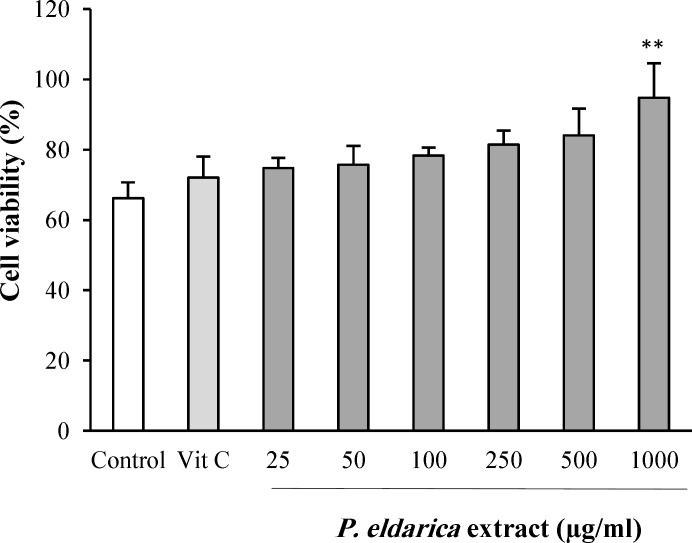
The effect of *P. eldarica* extract on proliferation of HUVECs. Cells were incubated with different concentrations of* P. eldarica* extract (25-1000 µg/ml) or vitamin C (100 µg/ml) for 24 h. The cell viability was determined and compared with the control (untreated cells) by the MTT assay. Values are mean±SEM from three independent experiments in triplicate. ^**^P<0.01 versus control (untreated cells

The probable cytotoxicity of *P. eldarica* extract on HUVECs was evaluated by MTT assay. There was no inhibitory effect on HUVECs viability after exposure to *P. eldarica* extract (25-1000 µg/ml) for 24 h (Fig. 1). Interestingly, *P. eldarica* extract at the concentration of 1000 µg/ml increased the viability of HUVECs.

**Cytoprotective effect of** ***P. eldarica*** **extract against H**_2_**O**_2_**-induced oxidative stress **

**Fig. 2:**
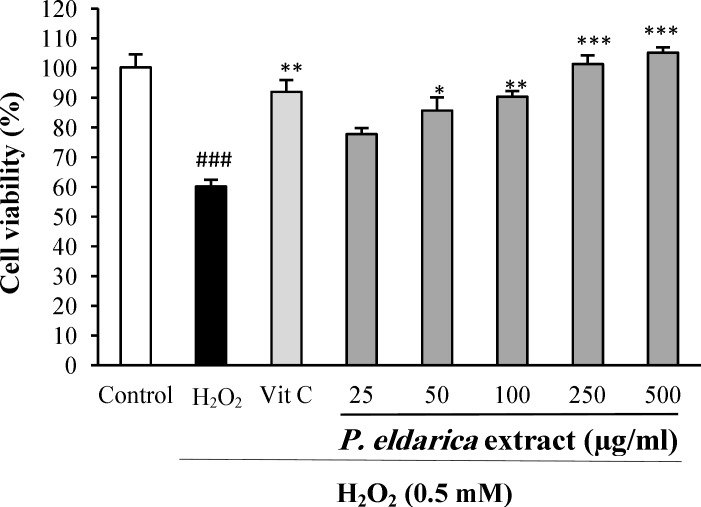
The effect of *P. eldarica* extract on H_2_O_2_-induced oxidative stress in HUVECs. Cells were incubated with H_2_O_2_ (0.5 mM, 2 h) after pretreatment with different concentrations of* P. eldarica* extract (25-500 µg/ml) or vitamin C (100 µg/ml). The cell viability was determined by the MTT assay. Values are mean±SEM from three independent experiments in triplicate. ^###^*P*<0.001 versus control (untreated cells),^ *^P<0.05, ^**^P<0.01 and ^***^P<0.001 versus H_2_O_2_-stimulated cells

When the HUVECs were exposed to oxidative damage induced by H_2_O_2_ at 0.5 mM for 2 h, a significant drop was observed in cell viability (*P*<0.001). Pretreatment of HUVECs with *P. eldarica* extract at the concentrations of 50-500 µg/ml significantly reduced the cytotoxicity effects of H_2_O_2_ (Fig. 2). 

**Effects of** ***P. eldarica*** **extract on intra- and extra-cellular hydroperoxides concentration**

**Fig. 3:**
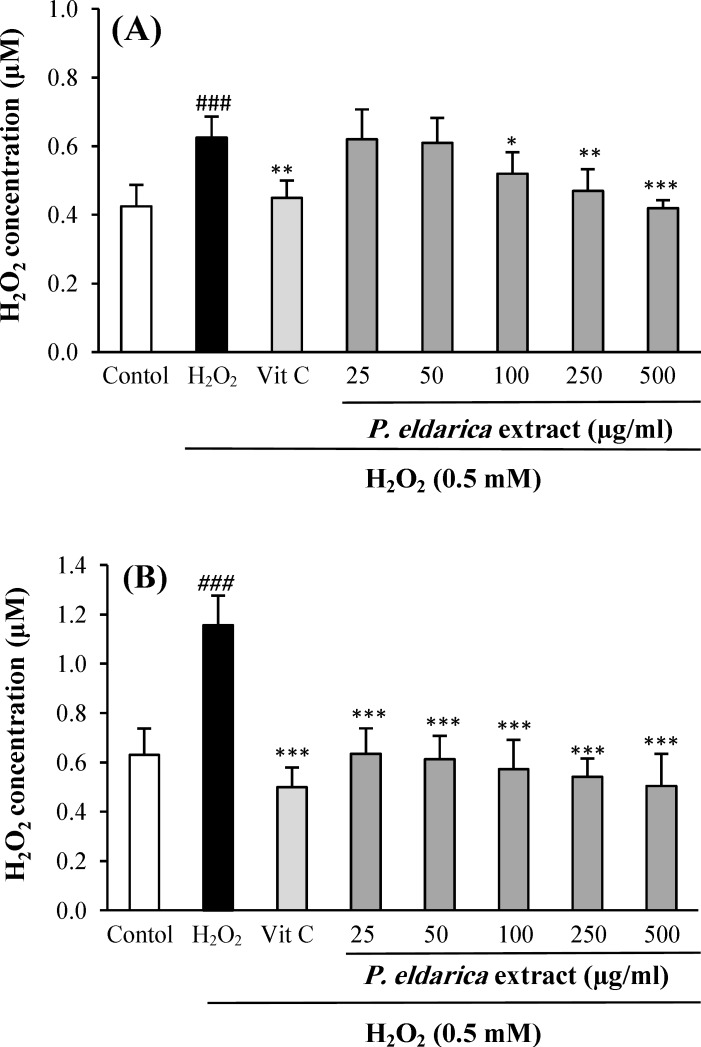
The effect of* P. eldarica* extract on intra-cellular (A) and extra-cellular (B) hydroperoxides concentration in HUVECs. Cells were incubated with H_2_O_2_ (0.5 mM, 2 h) after pretreatment with different concentrations of* P. eldarica* extract (25-500 µg/ml) or vitamin C (100 µg/ml). The hydroperoxides concentration was determined by FOX1 method. Values are mean±SEM from three independent experiments in triplicate. ^###^*P*<0.001 versus control (untreated cells),^ *^P<0.05, ^**^P<0.01 and ^***^P<0.001 versus H_2_O_2_-stimulated cells

Figure 3 show the effects of *P. eldarica* extract on intra- and extra-cellular hydroperoxides concentration in HUVECs after exposure to the oxidative stress induced by H_2_O_2_. The incubation of HUVECs with *P. eldarica* extract significantly decreased the intra-cellular hydroperoxides level at the concentrations of 100-500 µg/ml as compared with the control group. Pretreatment with* P. eldarica* extract also reduced the extra-cellular hydroperoxides level at the concentrations of 25-500 µg/ml (*P*<0.001).

**Effects of** ***P. eldarica*** **extract on cell-free and intra- and extra-cellular FRAP value **

**Fig. 4:**
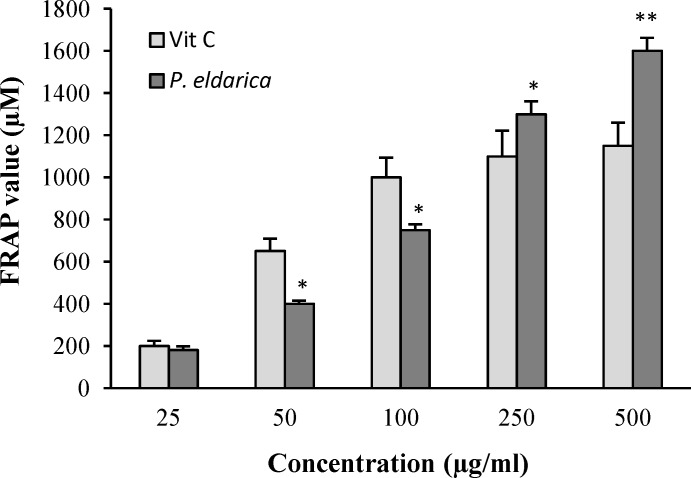
Ferric reducing antioxidant power (FRAP) values of different concentrations of* P. eldarica* extract and vitamin C (25-500 µg/ml). Values are means±SEM from three independent experiments in triplicate.^ *^P<0.05 and ^**^P<0.01 versus vitamin C group at the same concentration

**Fig. 5.**
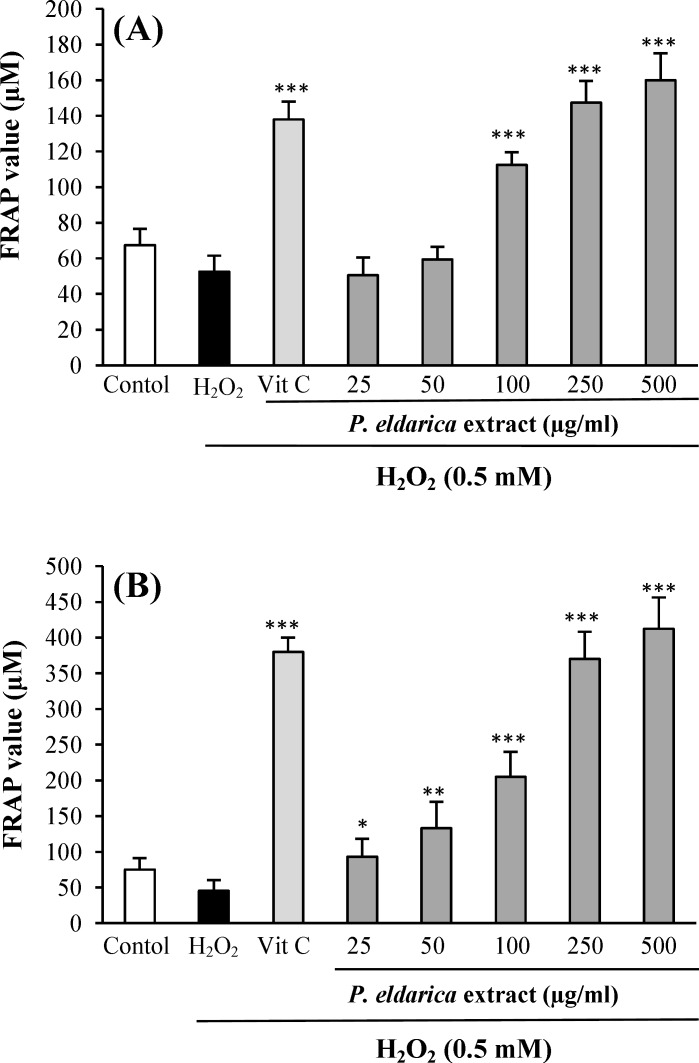
Effect of* P. eldarica* extract on intra-cellular (A) and extra-cellular (B) Ferric reducing antioxidant power (FRAP) value in HUVECs. Cells were incubated with H_2_O_2_ (0.5 mM, 2 h) after pretreatment with different concentrations of* P. eldarica* extract (25-500 µg/ml) or vitamin C (100 µg/ml). Values are means±SEM from three independent experiments in triplicate. ^***^P<0.001 versus H_2_O_2_-stimulated cells

The FRAP value of *P. eldarica* extract without cell and also in intra- and extra-cellular fluids was evaluated. An increasing trend in FRAP value was observed with increasing *P. eldarica* extract concentrations in a cell-free assay (Fig. 4). The incubation of HUVECs with *P. eldarica* extract significantly increased the FRAP levels in the intra-cellular fluid at the concentrations of 100-500 µg/ml (Fig. 5A) and in extra-cellular fluid at the concentrations of 25-500 µg/ml (Fig. 5B). 

## DISCUSSION

The present study demonstrated the protective effect of *P. eldarica* extract against H_2_O_2_-induced toxicity in HUVECs at the concentration range of 25-500 µg/ml.

Also, this herbal extract did not show any cytotoxic effects at the concentration range of 25-1000 µg/ml. *P. eldarica* extract decreased hydroperoxides concentration and increased FRAP value in intra- and extra-cellular fluid at the concentration ranges of 100-500 µg/ml and 25-500 µg/ml, respectively. 

Endothelial cells have a crucial role in the regulation of vascular physiological functions^[^^26^^]^. Numerous studies have indicated the role of oxidative stress in development of endothelium dysfunction^[^^27^^]^. H_2_O_2_, a non-free radical with oxidative ability, is widely used as a template substance to induce oxidative stress and apoptosis in various cell types such as endothelial cells^[^^28^^]^. As a small molecule lacking electrochemical charge, H_2_O_2_ can easily pass through the cell membrane and act as an intracellular second messenger in some vascular processes, including remodeling, inflammation, growth and apoptosis^[^^29^^]^.

During the vascular pathological conditions, nicotinamide adenine dinucleotide phosphate oxidase, xanthine oxidase and uncoupled endothelial NO synthase are the main producers of H_2_O_2_^[^^30^^,^^31^^]^. After the exposure of endothelial cells to H_2_O_2_, inflammatory responses including increased expression of intercellular adhesion molecule-1, platelet activating factor, and P-selectin, upregulation of monocyte chemoattractant protein-1, activation of NF-κB and also neutrophil adhesion to endothelium occur^[^^32^^]^.

In the present study, HUVECs exposure to H_2_O_2 _(0.5 mM) induced cell growth suppression and significantly raised the intra- and extra-cellular hydroperoxides levels assessed by FOX-1 method, which is a sensitive method for estimation of hydroperoxides^[^^33^^]^. Oxidative stress induced by H_2_O_2 _also reduced total antioxidant capacity. Several studies have reported decreased antioxidant capacity, such as superoxide dismutase, catalase and glutathione peroxidase, and vitamins C and E in CVDs^[^^34^^,^^35^^]^.

Pretreatment of HUVECs with *P. eldarica* extract significantly reduced the hydroperoxides level and increased FRAP; however, the extract provided less intra-cellular protection at low concentrations. Pinaceae is one of the largest families of conifers. The genus *Pinus* consists of various evergreen and aromatic trees as true pines, which is widely spread in many countries including Iran^[^^36^^]^. *P. eldarica*, as one of the most common pines in Iran composed of different parts, including needles, buds, nuts, and resin, has been commonly used in traditional medicine^[^^16^^]^. The high amounts of phenolic compounds, including catechin, ferulic acid, caffeic acid, and taxifolin have been identified in *P. eldarica* bark extract^[^^19^^]^. There are also high contents of monoterpenes and sesquiterpenes in composition of the bark oil of the *P. eldarica*^[^^19^^]^. Phenolic compounds are natural chemicals consisting of hydroxyl groups and possess strong antioxidant properties because of their reactivity with radical species and chelating metal ions^[^^37^^]^. The favorable cardiovascular effects of phenolic compounds have been shown in various studies^[^^1^^,^^11^^]^. The prevention of vascular oxidative stress and consequently the prevention of endothelial dysfunction has been reported in prediabetic rats following catechin intake^[^^38^^]^. Caffeic acid and ferulic acid belonging to cinnamic acid derivatives can reduce the risk of cardiovascular disorders by inhibiting the production of ROS^[^^39^^]^. Terpenoids are also plant antioxidants with helpful effects on cardiovascular system, including vasorelaxation as well as decreasing blood pressure and heart rate^[^^40^^]^.

In summary, this study showed the cytoprotective and antioxidant effects of hydroalcoholic extract obtained from the stem bark of *P. eldarica* in oxidative stress conditions in HUVECs. Regarding the high content of phenolic compounds,* P. eldarica *extract could be a good antioxidant candidate for improving endothelial function in exposure to oxidative stress conditions. 
